# Repurposing Drugs in Oncology (ReDO)—mebendazole as an anti-cancer agent

**DOI:** 10.3332/ecancer.2014.443

**Published:** 2014-07-10

**Authors:** Pan Pantziarka, Gauthier Bouche, Lydie Meheus, Vidula Sukhatme, Vikas P Sukhatme

**Affiliations:** 1 Anticancer Fund, Brussels, 1853 Strombeek-Bever, Belgium; 2 The George Pantziarka TP53 Trust, London KT1 2JP, UK; 3 GlobalCures, Inc, Newton, MA 02459, USA; 4 Beth Israel Deaconess Medical Centre and Harvard Medical School, Boston, MA 02215, USA

**Keywords:** drug repurposing, anti-helminthic, metronomic chemotherapy, cancer, ReDO Project

## Abstract

Mebendazole, a well-known anti-helminthic drug in wide clinical use, has anti-cancer properties that have been elucidated in a broad range of pre-clinical studies across a number of different cancer types. Significantly, there are also two case reports of anti-cancer activity in humans. The data are summarised and discussed in relation to suggested mechanisms of action. Based on the evidence presented, it is proposed that mebendazole would synergise with a range of other drugs, including existing chemotherapeutics, and that further exploration of the potential of mebendazole as an anti-cancer therapeutic is warranted. A number of possible combinations with other drugs are discussed in the Appendix.

## Introduction

Mebendazole (MBZ) is a broad-spectrum benzimidazole anti-helminthic drug, in the same class as albendazole, flubendazole, oxfendazole, and others. It is commonly prescribed to treat a range of parasitical worm infections, including threadworm, tapeworms, roundworms, and other nematode and trematode infections in humans and domestic animals. MBZ is available as a generic drug; common trade names have included Vermox (Janssen Pharamceutica) and Ovex (McNeil Products Ltd) in the US and Europe. It is generally available over the counter in European countries, but the last US manufacturer, Teva Pharmaceuticals, ceased production at the end of 2011, although the drug retains US Food and Drug Administration (FDA) approval. It is available in the US from compounding pharmacies, for example, Pavillion Compounding Pharmacy in Atlanta.

## Dosage

For human use, the most common formulation of MBZ is as 100 mg chewable tablets. The dosage varies according to the type of helminthic infection being treated. Pinworms are treated with a single 100 mg treatment, whereas roundworms or hookworms are treated with 100 mg twice a day for three days. MBZ, along with albendazole, is also used on a long-term basis for the treatment of human cystic and alveolar echinococcosis (also known as hydatid disease). According to the guidelines published by the World Health Organisation (http://whqlib-doc.who.int/bulletin/1996/Vol74-No3/bulletin_1996_74%283%29_231-242.pdf), long-term treatment of cystic echinococcosis using MBZ is at a dosage of 40–50 mg/kg/day for at least 3–6 months. For alveolar echinococcosis, the dose is 40–50 mg/kg/day, with treatment for at least two years, and possibly longer for patients with inoperable disease. Indeed, there are documented cases of treatment periods of ten or more years [1, 2].

## Toxicity

MBZ has low toxicity, though patients may suffer from transient symptoms, such as abdominal pain and diarrhoea in cases of massive infection and excretion of parasites. Hypersensitivity reactions, such as rash, urticaria, and angioedema, have been observed on rare occasions. MBZ is contraindicated during pregnancy. Caution is also recommended in treating infants below the age of 2, primarily due to a lack of data in such cases [3].

In the case of long-term administration of MBZ for echinococcosis, the evidence is that, in general, the treatment is well tolerated, but the specific treatment for some patients has to be discontinued. For example, in one open-labelled observational study, the patients treated with MBZ for alveolar echinococcosis (average: 24 months) experienced few adverse reactions, and in only three patients (of 17), the treatment was changed to albendazole due to intolerable side effects (reversible alopecia, psychological disturbance, and drop in performance) [4].

## Pharmacokinetics

First-pass metabolism of MBZ ensures that only about 20% of the oral dose reaches systemic circulation, with maximum plasma concentration reached 2–4 hours post-administration. Dosing with a high-fat meal is known to modestly increase bioavailability [5]. Chronic dosing of MBZ increases plasma concentration by a factor of between two and three compared to single dose [3, 6]. In one series of patients treated with chronic MBZ at a dose of 40 mg/kg/day for hydatid disease, the mean peak plasma level was 137.4 ng/ml [0.47 μM] after a single dose of 10 mg /kg; however, there was high inter-patient variability (99.4–500 ng/ml [0.34–1.69 μM]). For patients not on chronic treatment, an initial treatment of MBZ at the same dose produced a mean peak plasma level of 69.5 ng/ml [0.24 μM], (17.5–116.2 ng/ml [0.06–0.39 μM]) [6].

The poor bioavailability has long been recognised, and strategies to improve this remain actively researched, these strategies have included alternative formulations with vegetable oils [7–9], altering the crystalline structure of MBZ [10] and investigations into PEGylation [11].

Albendazole and MBZ interact with cimetidine, which inhibits metabolism and has been documented to increase MBZ plasma levels, (maximum serum levels rose to 82.3 ± 41.8 ng/ml [0.28 ± 0.14 μM ] from 55.7 ± 30.2 ng/ml [0.19 ± 0.10 μM], on 1.5 g of MBZ following chronic dosing of cimetidine at 400 mg three times a day for 30 days) [12]. This may be an important interaction with clinical relevance in that it suggests a strategy to increase bioavailability should that be required to increase the anti-cancer effect. Given that cimetidine may also have some anti-cancer activity [13], it also suggests that an investigation into possible synergies with MBZ over and above the effect on bioavailability would be an interesting avenue to explore.

High intra- and inter-patient variability may be an important factor in assessing response to MBZ as a possible anti-cancer therapeutic. However, it is clear that plasma levels achieved by chronic and high-dosing schedules are in the range necessary for clinical activity based on the pre-clinical evidence assessed in the following section.

## Pre-clinical evidence in cancer—*in vitro* and *in vivo*

In 2002, Mukhopadhyay and colleagues showed that MBZ induced a dose- and time-dependant apoptotic response in a range of lung cancer cell lines [14, 15], with an IC50 of ~0.16 μM. Cells were arrested in the G2-M phase before undergoing apoptosis. Just as importantly, MBZ had no effect on normal HUVECs or WI38 fibroblasts, even at a concentration of 1 μM. Overall, the *in vitro* results showed that MBZ inhibited lung cancer cell growth 5-fold compared to controls. Additionally, the authors confirmed the growth inhibitory effects of MBZ against breast, ovary, colon carcinomas, and osteosarcoma, producing IC50s that varied from 0.1 to 0.8 μM.

To test the *in vivo* response to the MBZ treatment, *nu/nu* mice were inoculated with subcutaneous injections of H460 non-small cell lung cancer cells [14]. Animals with established tumours (~3 mm diameter) were treated with 1 mg orally of MBZ every other day. Treated animals showed a dose-dependent arrest in tumour growth. The experiment was also repeated with C3H mice and the K1735 mouse cell line, and MBZ inhibited tumour growth in this syngeneic mouse model also. Mice treated with MBZ showed no side effects. Finally, the investigators also assessed whether MBZ might inhibit the formation of lung metastases and injected A549 cells into the tail vein of mice. In untreated controls, approximately 300 metastatic colonies appeared in the lungs by 21 days. Mice treated with 1 mg of MBZ every other day showed a mean colony count 80% lower than controls. Treatment with the established anti-microtubule agent paclitaxel showed no such reduction in colony formation.

Further pre-clinical evidence of MBZ anti-cancer activity was shown in adrenocortical cancer in 2008 [16], both *in vitro* and *in vivo*. H295R, SW-13 and WI-38 (normal fibroblast) cells lines were treated with different concentrations of MBZ *in vitro*, and the two cancer cells lines showed dose-dependent growth arrest, with IC50 of 0.23 μM for H295R and 0.27 μM for SW-13 cells, with no effect on the normal fibroblast cells. Tumour spheroid inhibition was tested against a dose of 1 μM of MBZ, which completely disaggregated the tumour spheroids and killed all cancer cells in about 20 days.

*In vivo* treatment of athymic nude mouse models of adrenocortical cancer showed that treatment with 1 mg and 2 mg MBZ significantly inhibited tumour growth in both implanted adrenocortical cancers. While there was little difference between the response of the primary tumours to 1 mg and 2 mg doses, the latter dose inhibited the formation of metastases from 50% of controls to 75%. No side effects were noted in the treated animals. Of note, a dose of 1 mg/day in a mouse weighing 20 gm corresponds to a human dose of approximately 500 mg daily for a 70 kg person, if extrapolated by surface area.

In 2008, the *in vitro* activity of MBZ against chemoresistant melanoma cell lines was assessed by Doudican *et al* [17]. A screening of 2000 small molecules against melanoma cells lines picked out ten compounds that had inhibitory action against the M-14 and SK-Mel-19 chemoresistant melanoma cells lines, but were non-toxic to normal melanocytes. Of these ten compounds, four were benzimidazoles —mebendazole, albendazole, fenbendazole, and oxybendazole—and of these four compounds, MBZ was selected for more detailed analysis based on its relative lack of toxicity and well-characterised pharmacokinetics. MBZ was shown to induce dose-dependent apoptosis in both cell lines with an average IC50 of 0.32 μM, while the equivalent for the non-cancerous melanocyte cell line was IC50 of 1.9 μM. MBZ also had the greatest inhibitory effect against the melanoma cells of the four benzimidazoles tested.

Subsequently, MBZ was shown to inhibit human melanoma xenograft growth in athymic female nude mice fed 1 mg or 2 mg oral MBZ every other day [18]. Tumour growth was reduced by 83% for the 1 mg dose and 77% for the 2 mg in comparison to controls. This was comparable to the growth inhibitory activity of 100 mg/kg of temozolomide (TMZ) by an intraperitoneal injection for 5 days, used as a positive control as it represents a well-characterised option for melanoma treatment. These results showed that oral MBZ produced equivalent responses to high-dose TMZ, but with no observed side effects.

MBZ activity in glioblastoma multiforme (GBM) was discovered serendipitously in 2011 by investigators, who observed that GBM xenografts were failing after mice models were fed albendazole to fight a spate of pin worm infections [19]. Further investigation showed that both albendazole- and MBZ-induced apoptosis in two GBM cells lines *in vitro* and *in vivo*. The *in vitro* IC50 of MBZ was 0.24 μM in the GL261 mouse glioma line, and 0.1 μM in the 060919 human GBM. *In vivo* results showed that oral MBZ treatment significantly extended mean survival up to 63% in syngeneic and xenograft orthotopic mouse glioma models.

Screening of compounds for activity against colon cancer cell lines also identified MBZ as a candidate molecule in work by Nygren and colleagues [20]. The authors set out to screen 1600 existing drugs for activity against two well-established colon cancer cell lines (HCT 116 and RKO) and found 64 candidate drugs, including a cluster of benzimidazoles (albendazole, mebendazole, oxybendazole and fenbendazole). Of these, further analysis was performed on MBZ and albendazole because, in the words of the authors, ‘they are registered pharmaceuticals for clinical use in humans, thus easily accessible for clinical testing’.

Diagnosis-specific activity was assessed using the NCI 60 z score data, which showed a high level of activity against leukaemia, colon cancer, CNS and melanoma panels of cell lines, with lesser activity in breast, ovarian, renal and NSCLC lines. It should be noted that the leukaemia panel had the highest level of sensitivity to MBZ, a finding that has not been further investigated to date. In the colon cancer panel, 80% of cells lines were sensitive to MBZ. Detailed *in vitro* treatment against five colon cancer cell lines (HCT 116, RKO, HT29, HT-8 and SW626), showed that all displayed IC50 of <5 μM, whereas the drug was largely inactive in the non-malignant cell lines.

Some work on *in vitro* efficacy against a chemoresistant breast cancer cell line (SKBr-3) was performed by Coyne and colleagues in 2013 [21]. A range of benzimidazoles, including MBZ and albendazole, were tested and found to cause significant growth arrest and apoptosis, with flubendazole and MBZ showing the greatest level of cytotoxic activity. MBZ reduced cell survival by 63.1% at a dose of 0.5 μM.

Finally, Schmit showed that a range of benzimidazoles, including MBZ, possess anti-neoplastic activity against the DS 17 canine osteosarcoma cell line *in vitro* [22]. Canine osteosarcoma is an excellent animal model of the human disease. The results obtained showed that MBZ induced cell cycle arrest and apoptosis at MBZ doses that are clinically achievable with oral dosing.

## Human data in cancer

No clinical trials of MBZ as a cancer treatment have been completed to date. However, there are two well-documented case reports in the literature in favour of re-purposing MBZ as an anti-cancer therapy.

In 2011, a case of long-term tumour control in metastatic adrenocortical cancer was published [23]. Adrenocortical cancer is a relatively rare malignancy with few treatment options in the case of non-resectable disease. The patient had experienced disease progression despite multiple chemotherapeutic protocols and several rounds of surgery. After all other treatment options had been exhausted, the patient discovered the pre-clinical evidence of MBZ action against adrenocortical cancer via Pubmed and forwarded the information to the clinicians, who agreed to use it based on this evidence and the relatively low toxicity of treatment. Monotherapy commenced with MBZ at a typical anti-helminthic dose of 100 mg twice a day. The patient experienced some regression in metastatic lesions, and overall the disease remained stable for 19 months of MBZ monotherapy, tolerating the treatment without side effects, and his quality of life returned to his baseline prior to his initial surgery. However, 24 months after the commencement of oral MBZ a scan showed disease progression, and everolimus was added to the MBZ but without additional benefit in disease control.

A case of metastatic colon cancer treated with MBZ was described by Peter Nygren and Rolf Larsson in 2013 [24]. Here, a 74-year-old patient suffering from progressive metastatic colon cancer had been treated first with capecitabine, oxaliplatin, and bevacizumab, and then by capecitabine and irinotecan in the face of disease progression, and who had no standard treatment options available was started on an oral dose of MBZ of 100 mg twice a day. MBZ was selected based on the author’s previous pre-clinical work with MBZ [20]. After six weeks of monotherapy, radiological evaluation showed near complete remission of metastatic lesions in the lungs and lymph nodes and a good partial remission in the liver. However, the patient experienced elevated liver enzymes (AST and ALT), so MBZ was temporarily stopped and then started at half the dose, with the patient reporting no ill effects. Liver enzymes normalised and a subsequent round of CT scans confirmed the initial disease response. After ceasing treatment for approximately three months, the patient developed brain metastases that were treated with radiotherapy, following by evidence of disease in the lymph nodes. MBZ treatment was not recommenced following the discovery of the brain metastases or in subsequent disease progression. A further five patients have been treated, with one experiencing a minor remission [Private communication from Peter Nygren].

## Clinical trials

There are currently two clinical trials of MBZ in cancer, both for brain tumours.

One is a Phase I open label study, at John Hopkins Hospital, of MBZ in newly diagnosed high-grade glioma patients receiving temozolomide (http://clinicaltrials.gov/ct2/show/NCT01729260). Patients recruited to the trial are treated on a 28 day cycle of 500 mg MBZ tablets three times a day. The primary end point is to determine the maximum tolerated dose of MBZ with temozolomide. A secondary end point is to determine if MBZ with current standard of care can slow tumour progression. Study completion is scheduled for November 2014 (at the time of writing in January 2014).

The other clinical trial is at Cohen Children’s Medical Centre of New York in paediatric patients with low-grade gliomas (http://clinicaltrials.gov/ct2/show/NCT01837862). This is a Phase I and II pilot study of MBZ in combination with vincristine, carboplatin, and temozolomide. The study design is non-randomised and open label, with comparison between standard of care and standard of care plus MBZ arms. The MBZ dose is 100 mg twice a day over the 70 weeks of treatment. The primary objective of the Phase I part of the trial is to determine if the standard dose of MBZ 100 mg twice daily is ‘well-tolerated’ when used in combination with the current three-drug regimen. At the end of Phase I potential subjects will be offered the chance to receive the three-drug regimen + MBZ for 70 weeks, or else to enrol as part of the control group receiving the three-drug regimen alone. For the Phase II portion of the study, the outcome variables of interest are progression-free and overall survival. Study completion is scheduled for December 2017 (as of December 2013).

## Mechanism of action

The anti-parasitic action of MBZ is due to its action as a microtubule-disrupting agent acting to prevent the polymerisation of tubulin in the gut of helminths, causing the parasites to die [25]. Tubulin is vital to cell division and is therefore a cancer target for several widely used chemotherapy drugs, including paclitaxel, colchicine, and vincristine. MBZ, as with the other benzimidazoles, binds to the colchicine-binding domain of tubulin [26].The inhibition of tubulin polymerisation by MBZ has been confirmed *in vitro* in a glioblastoma model [19] and in a melanoma model [17]. The latter work suggested that the apoptotic response to microtubule disruption is mediated by Bcl-2 phosphorylation. Subsequent work on melanoma confirmed this result, and also showed that MBZ decreased the levels of X-linked inhibitor of apoptosis (XIAP) [18], but to date this has not been confirmed in non-melanoma cell lines.

While there are rare reports of reversible alopecia, urticaria, rash, gastro-intestinal upset, leukopenia, and neutropenia in some patients treated with high-dose MBZ, all adverse effects associated with other microtubule disruption agents, there do not appear to be any reports of peripheral neuropathy, which is commonly considered a classic adverse effect of microtubule disrupting agents, including the taxanes and the vinca alkaloids [27]. While this may suggest that the action of MBZ is independent of microtubule disruption, it may also be related to the fact that MBZ acts via the colchicine-binding domain, and that like colchicine, there is little effect in terms of neuropathic pain [28]. Of course, it is also possible that the anticancer activity of MBZ is mediated by additional molecular targets yet to be elucidated.

MBZ appears to be effective through p53-dependent and independent pathways. For example, in lung cancer cell lines, it was found that MBZ treatment caused post-translational p53 stabilization and the downstream expression of p21 and MDM2 [14]. In p53-null lung cancer cells exposure to MBZ caused cytochrome c accumulation, activation of caspase-9 and caspase-8, and cleavage of PARP and procaspase-3. This independence of p53 status is also evident in the analysis of melanoma cells, where wild-type and mutant p53 cell lines were sensitive to MBZ [17].

There has been conflicting evidence regarding the effect that MBZ has on tumour neo-vascularisation, with some reports finding evidence that it has an anti-angiogenic effect and others finding none.

In the earliest work on the anti-cancer activity of MBZ, Mukhopadhyay and colleagues reported an anti-angiogenic effect on human lung cancer xenograft models [14]. However, *in vivo* analysis of adrenocortical cancer models failed to detect any anti-angiogenic activity compared to controls [16]. Some support for an anti-angiogenic action comes from an *in silico* study, which indicated that MBZ inhibits the action of VEGFR-2 by binding to it, a finding validated *in vitro* using a human umbilical vein endothelial cell (HUVEC) based angiogenesis functional assay [29]. Of note, the related drug albendazole has shown anti-angiogenic properties in an ovarian cancer model and in drug-resistant cell lines [30, 31], suggesting that an anti-angiogenic action may be common across a number of benzimidazoles.

To date, the effect of MBZ or other benzimidazole on the immune response in cancer has not been investigated, though there is some evidence that albendazole synergised to stimulate the cellular immune response in mice treated for alveolar echinococcosis with the immunotherapeutic agent liposomal muramyl tripeptide phosphatidylethanolamine (L-MTP-PE) used in the treatment of osteosarcoma [32]. There is also increasing evidence that existing microtubule disrupting agents used at low or metronomic doses, including the taxanes and vinca alkaloids, exert a positive immunomodulatory action that may help to reverse the immunosuppressive effect of cancer [33–36]. We can speculate, mechanistically, that some of this immunomodulatory action is related to microtubule dynamics. Therefore, there may be a similar effect with MBZ and other benzimidazoles, and this may also be a factor in the anti-cancer effects of these drugs.

## Our take

### Next steps

Based on the evidence summarised in Table 1, it is our contention that human clinical trials of mebendazole in a range of cancer types is warranted. The known pharmacokinetics, relatively low toxicity (even with extended high-dosing protocols), low cost, and strong pre-clinical evidence make this an ideal candidate for re-purposing. Currently, there are only two early phase clinical trials getting under way, both in glioma. In addition to these, the evidence suggests that candidate cancer types to take to human trial include:

- melanoma,
- non-small cell lung cancer,
- adrenocortical cancer, and
- colon cancer.

Additional cancer types, which should be further investigated in animal studies include:

- breast cancer,
- leukaemia, and
- osteosarcoma.

As with other anti-cancer agents, it is most likely that MBZ will be more effective in combination with other drugs or treatment modalities. It should be noted that the first two clinical trials are using MBZ with current standard of care treatment in glioma, which in this case means a combination protocol with other drugs, principally temozolomide. Given the primary putative mechanism of action—microtubule disruption—there are a number of additional agents that warrant investigation for synergy with MBZ, some of which are listed in the Appendix.

### Other options

Finally, improved efficacy may also be possible through improvements in the bioavailability of MBZ. As touched on previously, there is evidence that the combination of MBZ with cimetidine increases plasma levels of MBZ [12], potentially improving the therapeutic effect. An alternative means of increasing the bioavailability is through the liposomal encapsulation of MBZ. While this approach has not been explored in an oncological context, some work in this area have been done to enhance the anti-parasitic action of MBZ and other benzimidazole anti-helminthics, including one paper that explored the combined effect of a liposomal benzimidazole (albendazole) and cimetidine and reported a very significant increase in therapeutic effect (including a 75–94% reduction in biomass of the hydatid cysts and a significant increase in survival time) in an animal model [37]. It is possible that a similar approach could yield improvements in the anti-cancer effect of MBZ.

### New protocols

Adding MBZ to the existing standard of care protocols, as the first two clinical trials have done, provides an opportunity to test whether there are incremental improvements in outcomes compared to the standard of care alone. However, we should also seek opportunities to create new protocols that combine MBZ with other repurposed drugs with similar low toxicity and potential anti-cancer activity. The intention is to create novel treatment options that are multi-targeted and which present minimal risk of toxicity. Of necessity, given our current state of knowledge, the combinations proposed in the supplementary material are speculative and informed primarily by mechanistic considerations and pre-clinical data. Additional pre-clinical studies are required, but given the urgency of unmet patient needs and the low toxicity of the proposed combinations, it may be argued that small patient trials or even off-label usage may also be warranted.

## Conclusion

The evidence for an anti-cancer effect of mebendazole treatment comes from *in vitro*, *in vivo*, *in silico*, and human data. Mechanistically, the microtubule action is well characterised in the laboratory and provides a similar rationale to some of the major classical chemotherapeutic drug classes, such as the taxanes and vinca alkaloids. With well-established pharmacokinetics and an excellent toxicity profile, this low-cost agent is a strong candidate for drug repurposing as an oncological treatment, both in combination with the existing standard treatments and alongside other candidate repurposing agents in a number of specific cancer types. We have outlined a number of these multi-drug combinations in the hope that clinicians can act upon this information to initiate clinical trials as a matter of some urgency.

## Figures and Tables

**Table 1.** A summary of pre-clinical evidence by cancer type.

Cancer Type	In Vitro	In Vivo	Case Report/Trial
**Adrenocortical**	[16]	[16]	[23]
**Breast**	[14, 21, 20]		
**Colon**	[14, 20]	[14]	[24]
**Glioma**	[19]	[19]	NCT01729260, NCT01837862
**Leukaemia**	[20]		
**Lung**	[15]	[15]	
**Melanoma**	[17, 20]	[18]	
**Osteosarcoma**	[14, 22]		
**Ovary**	[14, 20]		

**Table A1.** Proposed drug combinations with MBZ for specific indications.

Disease	Targets	Drug Combination
**Malignant Melanoma**	Microtubule disruption, inhibition of autophagy, anti-angiogenic and immunomodulation	Hydroxychloroquine *(NCT00962845)* Diclofenac or Celecoxib [24] Oral cyclophosphamide [39]
**NSCLC**	Microtubule disruption, AMPK/mTOR, Hedgehog signalling, COX-2 inhibition	Metformin *(NCT01997775)* Itraconazole [40] Diclofenac or Celecoxib *(NCT00520845)*
**Adrenocortical Carcinoma**	Microtubule disruption, anti-angiogenic, Hedgehog signalling	Itraconazole Oral cyclophosphamide [41]
**Glioblastoma Multiforme**	Inhibition of autophagy, microtubule disruption, Hedgehog pathway inhibition, anti-angiogenic	Hydroxychloroquine *(NCT00224978)* Itraconazole
**Colorectal Carcinoma**	Microtubule disruption, AMPK/mTOR, immunomodulation, anti-histamine, COX-2	Metformin *(NCT01941953)* Cimetidine [42] Diclofenac Oral vinorelbine [43]
**Osteosarcoma/ Soft-tissue Sarcoma**	Microtubule disruption, AMPK/mTOR, IGF-I, Hedgehog pathway inhibition, tumour vascularity, anti-angiogenic	Metformin Itraconazole Losartan Oral cyclophosphamide [44]
**Acute Myeloid Leukaemia**	Microtubule disruption, induction of apoptosis	Albendazole or oral vinorelbine [45] Diclofenac
**Breast Cancer (ER+ invasive ductal carcinoma)**	Microtubule disruption, AMPK/mTOR, anti-angiogenic	Metformin *(NCT01929811)* Oral cyclophosphamide and/or oral vinorelbine (NCT00954135)
**Ovarian Carcinoma**	Ovarian Carcinoma	Metformin *(NCT02050009)* Itraconazole Diclofenac (NCT01124435)

Note that references to clinical trials or published papers are indicative of trials or case reports where the drug (or analogue) has been used for the specific indication.

## Appendix

### Introduction

The following drugs warrant further investigation in combination with mebendazole (MBZ), both in pre-clinical studies and potentially in clinical trials. These combinations listed in Table A1 have been selected on the basis of existing pre-clinical and clinical experience in each of the indications. In some cases, these combinations replicate existing protocols currently being tested in clinical trials, but substitute known and repurposed drugs for the newer and/or more toxic agents currently being investigated. All these proposed combinations are expected to display relatively low toxicity and use low cost and generally available agents.

### Higher-priority agents

The agents listed below have a high degree of clinical evidence of efficacy and are currently either in clinical use in oncology or are currently being investigated in clinical trials. They have been selected as potential agents to be used in combination with MBZ. Note that these drugs are not listed in order of priority.

- *Metformin*: There is pre-clinical evidence that metformin potentiates the action of existing microtubule disrupting drugs in a range of cancer types, including endometrial cancers and paediatric sarcomas [1–3]. Given the low toxicity of metformin and its potential as an anti-cancer agent, the combination with MBZ should be explored, both in animal models and potentially in small clinical trials.
- *Metronomic chemotherapy*: While there is intense interest in the area of metronomic chemotherapy using taxanes or vinca alkaloids, progress has been restricted because of a lack of oral formulations of many of these drugs, with the exception of oral vinorelbine. Where existing microtubule targeting drugs without oral formulations are used in metronomic settings, it is normally as a weekly infusion in combination with daily dosing of oral cyclophosphamide or capecitabine. A number of clinical trials using oral vinorelbine have reported both low toxicity and evidence of clinical benefit in advanced cancers [4, 5]. MBZ also offers the possibility of exploring daily oral dosing of a microtubule disrupting agent in combination with low dose oral chemotherapy drugs and other agents used in such protocols (e.g. celecoxib or other anti-inflammatory). It is theorised that one of the principal methods of action of metronomic chemotherapy is through inhibition of neo-angiogenesis, and that escape from angiogenic control is associated with treatment failure. The addition of MBZ with existing metronomic protocols may increase the anti-angiogenic effect of treatment and prolong the therapeutic benefit.
- *Taxanes or Vinca Alkaloids*: Combinations of microtubule targeting agents, for example, paclitaxel or docetaxel and vinorelbine, act synergistically, and there are numerous trials exploring multiple combinations of different microtubule agents [6]. Pre-clinical evidence shows that the benzimidazole flubendazole synergises with vincristine and vinblastine *in vitro* and *in vivo* [7]. Given that MBZ has such low toxicity in comparison to many existing microtubule agents, combination therapy of MBZ with taxanes or vinca alkaloid drugs would seem a promising avenue to explore in human trials. One prospect is to combine MBZ with oral vinorelbine, offering the prospect of dual oral microtubule disrupting drugs, with low toxicity, either in standard dosing of vinorelbine or at metronomic dosing of both agents.
- *Albendazole or other benzimidazole*: There is evidence that the different benzimidazoles vary in their molecular targets and that combining them may improve efficacy and reduce the risks of acquired resistance. While this approach has not been explored in a cancer setting, there is pre-clinical and clinical evidence that the combination of MBZ and albendazole is a more effective treatment in certain hard to treat parasitic conditions [8, 9]. There is also some *in vitro* and *in vivo* evidence where albendazole exerts an anti-angiogenic action by down-regulating vascular endothelial growth factor (VEGF), an effect mediated through inhibition of tumoural hypoxia inducible factor (HIF-1α) [10]. As HIF-1α is implicated in multi-drug resistance in cancer, the combination of MBZ and albendazole warrants further investigation in drug-resistant tumours.
- *Itraconazole*: The anti-fungal drug itraconazole has shown some evidence of having anti-cancer activity, possibly through an anti-angiogenic action and inhibition of Hedgehog signalling pathway [11, 12]. It is currently being investigated in a number of clinical trials, principally in metastatic prostate cancer (e.g. www.clinicaltrials.gov/ct2/show/NCT00887458). A recently completed Phase II trial in basal cell carcinoma showed that in eight previously untreated patients with multiple tumours, four showed partial response and four had stable disease. In contrast, patients previously treated with vismodegib showed no significant changes in proliferation or tumour size [13]. There is laboratory evidence that Hedgehog inhibition can reverse resistance to taxane chemotherapy in a range of cell lines, including ovarian [14] and prostatic cancer [15]. There is specific evidence that itraconazole itself is able to reverse multi-drug resistance in resistant HeLa cells, at least *in vitro*, at doses achievable in humans [16].
- *Cimetidine*: The H2 receptor antagonist cimetidine, primarily used to treat peptic ulcers and heartburn, has shown *in vitro* and *in vivo* anticancer activity in a range of cell types and animal models, with a number of possible methods of action [17], including favourable impact on the immune system. A recent Cochrane Review suggested that there was a trend towards improved survival outcomes when cimetidine is used as perioperative and/or adjuvant treatment for early-stage colorectal cancer [18]. As was mentioned previously, there is also evidence that cimetidine can increase the plasma levels of MBZ [19]. A combination of cimetidine and MBZ would therefore be of interest, particularly in colorectal cancer where there is human evidence that each agent has some anticancer activity.
- *Diclofenac*: The non-steroidal anti-inflammatory drug (NSAID) is a commonly used anti-inflammatory analgesic with known activity as a COX-2 inhibitor, and is available both in topical and oral form. While there is evidence that perioperative or intraoperative diclofenac may be associated with lower risk of cancer recurrence or metastases following surgery [20], there are also active investigations of its direct anticancer role. In particular, diclofenac has shown pre-clinical activity in a range of cancer types, with *in vivo* evidence in melanoma [21] and ovarian cancer [22] in particular. Clinically, diclofenac has been used in the treatment of recurrent or aggressive desmoid tumours, including cases where diclofenac was combined with the microtubule targeting drug vinblastine [23]. Celecoxib, another COX-2 inhibitor, has also been used in combination with metronomically dosed paclitaxel in metastatic melanoma and shown some evidence of clinical activity [24]. Given the pre-clinical evidence of MBZ activity against melanoma and ovarian cancer and the similar evidence for diclofenac, early phase human trials of a combination treatment would be warranted.
- *Chloroquine/Hydroxychloroquine*: The anti-malarial drugs chloroquine and hydroxychloroquine are under active investigation in a range of clinical trials for cancer in combination with radiotherapy and/or alongside existing chemotherapy regimens. The putative mechanism of action of chloroquine is that it acts as an inhibitor of autophagy, acting therefore to restrict the ability of cancer cells to move to an autophagic state such that they move into apoptosis in response to cellular stresses initiated by chemotherapy or radiotherapy [25]. A particular focus of much pre-clinical and clinical research with chloroquine and hydroxychloroquine is in glioblastoma, with initial results from one small clinical trial showing a tendency to longer overall survival, though the result of the small sample size is not statistically significant [26]. The rationale for a combination of MBZ and chloroquine/hydroxychloroquine is stronger for those indications where the level of evidence for each agent is stronger, that is in glioblastoma and melanoma, and therefore human trials in these cancer types are warranted.
- *Clarithromycin*: A well-established macrolide antibiotic, clarithromycin has been used in an oncological setting for the eradication of Helicobacter Pylori infection or as a treatment for treatment-associated mycobacterial infection. It has also been used in combination therapy with lenalidomide, and dexamethasone for the treatment of multiple myeloma [27] or as a monotherapy for B cell lymphoma [28]. One mechanism of action that is being actively explored in relation to the anticancer effects of clarithromycin is a suggested action as an inhibitor of autophagy [29]. The role of autophagy in cancer is a complex one, but there is evidence that the inhibition of autophagy in established tumours abrogates a key survival mechanism utilised by cancer cells to protect themselves from a range of cancer treatments, including chemotherapy, radiotherapy and targeted therapies [30]. There are a number of clinical trials combining the autophagy inhibitor chloroquine with a range of chemotherapeutic drugs, including microtubule disrupters, in different cancers. The combination of MBZ and clarithromycin would mirror the underlying strategy of some of these existing trials but using low-cost and low-toxicity agents. This would be an approach most warranted in gliomas, where it has been shown that the inhibition of autophagy with chloroquine in addition to standard treatment improved survival [26].

### Other agents

The drugs listed below may also be suitable for combination treatments with MBZ and other agents, however, the evidence is not as strong and therefore this list must be viewed as more speculative.

- *2-Methoxyestradiol (2Me)*: A natural metabolite of estradiol, 2Me has shown promising anti-cancer activity in a number of clinical trials and is currently being developed as a drug under the trade name of Panzem (EntreMed Inc), with trials on-going in a range of solid tumours. Proposed methods of action include anti-angiogenesis, suppression of microtubule dynamics and inhibition of proliferation [31]. There have been a number of *in vitro* and *in vivo* studies assessing the synergistic action of 2Me with other microtubule-targeting drugs, including a recent study that assessed the combination of 2Me and albendazole in a xenograft colorectal cancer model and reported a significantly improved survival time [32].
- *Losartan*: The angiotensin II receptor antagonist losartan, used mainly to treat hypertension, is currently being investigated as a possible anticancer therapeutic, primarily for its role in counter-acting the reduced vascular perfusion caused by physical stresses within the tumour mass [33]. The direct role of angiotensin II in cancer progression, particularly with regards to the up-regulation of angiogenesis is also being investigated, and therefore losartan may also have an anticancer effect through its primary function as an angiotensin II receptor blocker (ARB) [34]. Tumour hypoxia and lack of vascular perfusion are often causes of treatment failure in chemotherapy, and therefore a possibility also with MBZ treatment. Therefore, a combination treatment of losartan and MBZ, particularly in solid tumours, such as osteosarcoma, would be of interest.
- *Omega 3 PUFAs*: There is pre-clinical evidence that omega 3 fatty acids eicosapentaenoic acid (EPA) and docosahexaenoic acid (DHA) can have chemosensitising effects in a range of cancer cell types and for a range of standard chemotherapeutic drugs [35]. There is also limited clinical evidence that oral supplementation with EPA/DHA can improve outcomes in colorectal and advanced non-small cell lung cancer [36, 37]. Given this sensitisation of tumours to chemotherapies, and the known improvement in MBZ bioavailability when taken with a fatty meal [38], the combination of EPA/DHA and MBZ should also be explored.
